# Identification of mine water sources using a multi-dimensional ion-causative nonlinear algorithmic model

**DOI:** 10.1038/s41598-024-53877-5

**Published:** 2024-02-08

**Authors:** Qiushuang Zheng, Changfeng Wang, Yang Yang, Weitao Liu, Ye Zhu

**Affiliations:** 1 School of Economics and Management, Beijing University of Posts and Telecommunications, Beijing, 100876 China; 2 College of Energy and Mining Engineering, Shandong University of Science and Technology, Qingdao, 266590 China; 3 State Key Laboratory of Mine Disaster Prevention and Control, Shandong University of Science and Technology, Qingdao, 266590 China

**Keywords:** Piper qualitative graphical method, R-factor dimensionality reduction, Water chemistry, Support vector machine, Water source ion identification, Hydrology, Engineering

## Abstract

Based on the nonlinear algorithmic theory, the R-SVM water source discrimination model and prediction method were established by using the piper qualitatively to compare the differences between the ionic components and R-type factor approximation indicator input dimensions. Taking the mine water samples of Zhaogezhuang Coal Mine as an example, according to the chemical composition analysis of the water samples from different monitoring points, six indexes of Na^+^, Ca^2+^, Mg^2+^, Cl^–^, SO_4_^2–^ and HCO_3_^–^ were selected as the discrimination factors. According to the water characteristics of each aquifer and the actual needs of discrimination, the water inrush sources in the mining area were divided into four categories: The goaf water is class I, Ordovician carbonate is class II, Sandstone fracture water from the 13 coal system is class III, and Sandstone fracture water from the 12 coal system is class IV. Taking 56 typical water inrush samples as training samples, 11 groups for prediction samples, establish the input index as typical ion content, output as water source type, using SPSS statistics and MATLAB to realize the R-SVM water source discriminant analysis model, automatically establishing the mapping relationship between the water quality indexes and the evaluation standards, which can achieve the purpose of rapid and accurate discrimination of the water sample data. The results showed that the accuracy of the R-SVM model classification was 90.90% in the verification of the water source discrimination example of Zhaogezhuang mine and the coupled model has high accuracy, good applicability and discriminant ability, and has certain guiding significance for the prevention and control of water damage and the related field work.

## Introduction

With the development of economy and society, the demand for mineral resources is steadily escalating. Mineral resources serve as the indispensable material foundation for human production activities^1–3^. Over the years, the development and utilization of mineral resources have necessitated a shift in mining focus, transitioning coal mines towards the extraction of intricate refractory mining bodies, such as deep orebody, broken soft orebody, alpine area orebody and low-grade orebody, and “three lower and one upper” ore bodies^4^. As mining intensity and depth increase, the extraction of mineral resources within complex geological structures becomes more challenging, giving rise to a surge in engineering predicaments. Among these challenges, mine water disasters emerge as a prominent threat to mining operations. Hence, the timely and precise identification of water source categories, constitutes essential prerequisites for averting water-related disasters and establishing a scientific foundation for swift rescue and management endeavors^5,6^.

Water chemistry data plays a crucial role in understanding the fundamental characteristics of aquifers and is vital for discriminating water sources^7^. Qualitative and quantitative methods are commonly employed to analyze water chemistry information for this purpose. Qualitative analysis, combined with water level dynamics, provides a rough determination of the syncline level. Piper's trilinear water chemistry analysis, on the other hand, is a convenient and visual tool for water quality classification and ion distribution^8^. The modified D-Piper trilinear diagram provides a solution for the challenge of visualizing ion distribution in large data sets^9^, leading to improved visualization and interpretation with an increase in data points. In addition, it is crucial to consider physicochemical information such as isotopes and radioactive elements in water bodies to reflect the essential characteristics and historical evolution of hydrogeology. The hydrogeochemical distribution, recharge sources, indicator tests, influencing factors, and evolutionary laws are analyzed based on conventional water chemistry, trace elements, and isotopes of the aquifer^10^. Gibbs’ semi-qualitative model^11^ is employed to analyze the hydration types of surface water and shallow groundwater, providing insights into the controlling factors, formation mechanisms, and recharge sources of isotopes in various aquifers. This analysis reveals the distinct weathering and hydration characteristics of different water bodies. However, qualitative methods alone face limitations in similar aquifers due to the ambiguous relationship between indicators, overlapping water quality characteristics, and unclear distribution boundaries^12^. To overcome these limitations, quantitative analysis^13^ is utilized to uncover the inherent laws of water chemistry data, establish mathematical models for determining water source types, elucidate the close connection between water quality indicators and determination criteria, and minimize the errors associated with qualitative analysis methods.Fisher function discrimination of water source locations based on fuzzy clustering and factor analysis^14,15^ and Bayes classification of water sources^16,17^ are employed to determine the water sources of sudden water in the mine area, with improved accuracy of discrimination. Groundwater is subject to multiple factors coupling due to the variability of mine geological structure, the complexity of hydrogeological characteristics, and the diversity of mining conditions, resulting in fuzzy connections and complex nonlinear relationships between water quality indicators and discriminatory criteria. However, model studies for index simplification through data dimensionality reduction are limited, and the redundancy of information between water chemical components reduces discriminative accuracy, requiring further optimization of the discrimination model.

This study addresses the water quality assessment system by introducing a novel approach that combines qualitative and quantitative analysis. A key contribution of this research is the utilization of Piper's trilinear diagram graphical method to analyze the variation pattern of ionic composition in aquifers and water chemistry characteristics through point mapping. By comparing the differences in ionic composition among aquifers and evaluating the proximity to the target water body, an initial classification of water quality is established.This fills the gap in existing research on risk factor internal information mining and machine learning, and provides a foundation for subsequent quantitative water source discrimination. To achieve this, a coupled discrimination model, integrating the R-factor and Support Vector Machine, is developed to uncover inherent characteristics within water chemistry data and automatically establish the mapping relationship between water quality indices and evaluation criteria. This innovative approach enables precise identification of water source types and provides valuable guidance for effective water damage control in practical engineering applications.

## Theoretical basis

### Principle of R-factor dimensionality reduction

There are m test variables $Z_{i} (i = 1,2,3, \cdots ,m)$, which may be correlated, and each $Z_{i}$ contains independently existing common factor $f_{j} \left( {j = 1,2, \cdots ,p} \right)$, $P \le m$ where $Z_{i}$ contains *m* mutually uncorrelated unique factors $u1,u2,u3, \cdots ,um$, and *u* and *f* are mutually uncorrelated. Each *Z* can be linearly characterized by *f* and *u* as^18^:

1 $$\left\{ {\begin{array}{*{20}l} {Z_{1} = a_{11} f_{1} + a_{12} f_{2} + \cdots + a_{,p} f_{p} + c_{1} u_{1} } \hfill \\ {Z_{2} = a_{21} f_{1} + a_{22} f_{2} + \cdots + a_{2p} f_{p} + c_{2} u_{2} } \hfill \\ \vdots \hfill \\ {Z_{m} = a_{m1} f_{1} + a_{m2} f_{2} + \cdots a_{np} f_{p} + c_{m} u_{m} } \hfill \\ \end{array} } \right..$$

Expressed as matrix:

2 $$\left( {\begin{array}{*{20}c} {Z_{1} } \\ {Z_{2} } \\ \vdots \\ {Z_{m} } \\ \end{array} } \right) = \left( {\begin{array}{*{20}c} {a_{11} } & {a_{12} } & \cdots & {a_{1n} } \\ {a_{21} } & {a_{22} } & \cdots & {a_{2n} } \\ \cdots & \cdots & \ddots & \cdots \\ {a_{m1} } & {a_{m2} } & \cdots & {a_{nn} } \\ \end{array} } \right)\left( {\begin{array}{*{20}c} {f_{1} } \\ {f_{2} } \\ {f_{3} } \\ {f_{4} } \\ \end{array} } \right) + \left( {\begin{array}{*{20}c} {c_{1} u_{1} } \\ {c_{2} u_{2} } \\ \vdots \\ {c_{m} u_{m} } \\ \end{array} } \right).$$

Abbreviated as:

3 $$Z = A \cdot F + C \cdot U.$$

The factor analysis method lies in replacing *Z* by* F* through Eqs. (2) and (3), conditioned on $p < m$, which can streamline the number of dimensions to reduce redundancy. The specific steps are^19^:

1. Construct sample matrix and perform correlation test,

Collect the p-dimensional random variable $X = (x_{1} ,x_{2} , \cdots x_{p} )^{T}$ and construct the sample matrix:

4 $$X = \left[ {\begin{array}{*{20}c} {x_{1}^{T} } \\ {x_{2}^{T} } \\ \vdots \\ {x_{n}^{T} } \\ \end{array} } \right] = \left[ {\begin{array}{*{20}c} {x_{11} } & {x_{12} } & \cdots & {x_{1p} } \\ {x_{21} } & {x_{22} } & \cdots & {x_{2p} } \\ \vdots & \vdots & \vdots & \vdots \\ {x_{n1} } & {x_{n2} } & \cdots & {x_{np} } \\ \end{array} } \right].$$

The KMO or Bartlett test was used to test the correlation of variables, and if the correlation coefficient is less than 0.3, there is no sense of dimensionality reduction. If the correlation is strong means that the commonality of variables can be extracted and is suitable for factor analysis.

(2) Processing to obtain the standardized matrix,

The standardization is done through the following:

5 $$Z_{ij} = \frac{{y_{ij} - \hat{y}_{j} }}{{s_{ij} }}(i = 1,2, \cdots ,p).$$

The standardized matrix is obtained:

6 $$Z = \left[ {\begin{array}{*{20}c} {z_{1}^{T} } \\ {z_{2}^{T} } \\ \vdots \\ {z_{n}^{T} } \\ \end{array} } \right] = \left[ {\begin{array}{*{20}c} {z_{11} } & {z_{12} } & \cdots & {z_{1p} } \\ {z_{21} } & {z_{22} } & \cdots & {z_{2p} } \\ \vdots & \vdots & {} & \vdots \\ {z_{n1} } & {z_{n2} } & \cdots & {z_{np} } \\ \end{array} } \right].$$

(3) Calculate the correlation matrix,

The correlation coefficient matrix is obtained as follows:

7 $$Z = \left[ {r_{ij} } \right]_{p \times p} = \frac{{Z^{T} Z}}{n - 1}.$$

In addition,

8 $$r_{j}^{2} = \frac{{\sum\limits_{i} = 1^{n} (z_{ij} - z_{j} )^{2} }}{n - 1}(i,j = 1,2, \cdots ,p).$$

The correlation calculation is performed on the standardized matrix *Z*. The eigenvector values of $|R - \lambda I_{P} | = 0$ are obtained based on the features of the correlation matrix, and then the common factors are extracted using the above approach, making the information utilization rate cover more than 85%.

(4) Calculate the factor load matrix, rotate the load matrix, and obtain the matrix *U*,
  9 $$U = \left[ {\begin{array}{*{20}c} {u_{1}^{T} } \\ {u_{2}^{T} } \\ {u_{3}^{T} } \\ {u_{4}^{T} } \\ \end{array} } \right] = \left[ {\begin{array}{*{20}c} {u_{11} } & {u_{12} } & \cdots & {u_{1p} } \\ {u_{21} } & {u_{22} } & \cdots & {u_{2p} } \\ \vdots & \vdots & {} & \vdots \\ {u_{n1} } & {u_{n2} } & \cdots & {u_{np} } \\ \end{array} } \right].$$

$u_{i}$ Principal component vector of the i sample. $u_{ij}$ Projection of the vector on the unit eigenvector.

### Support vector machine principle

**Figure 1:**
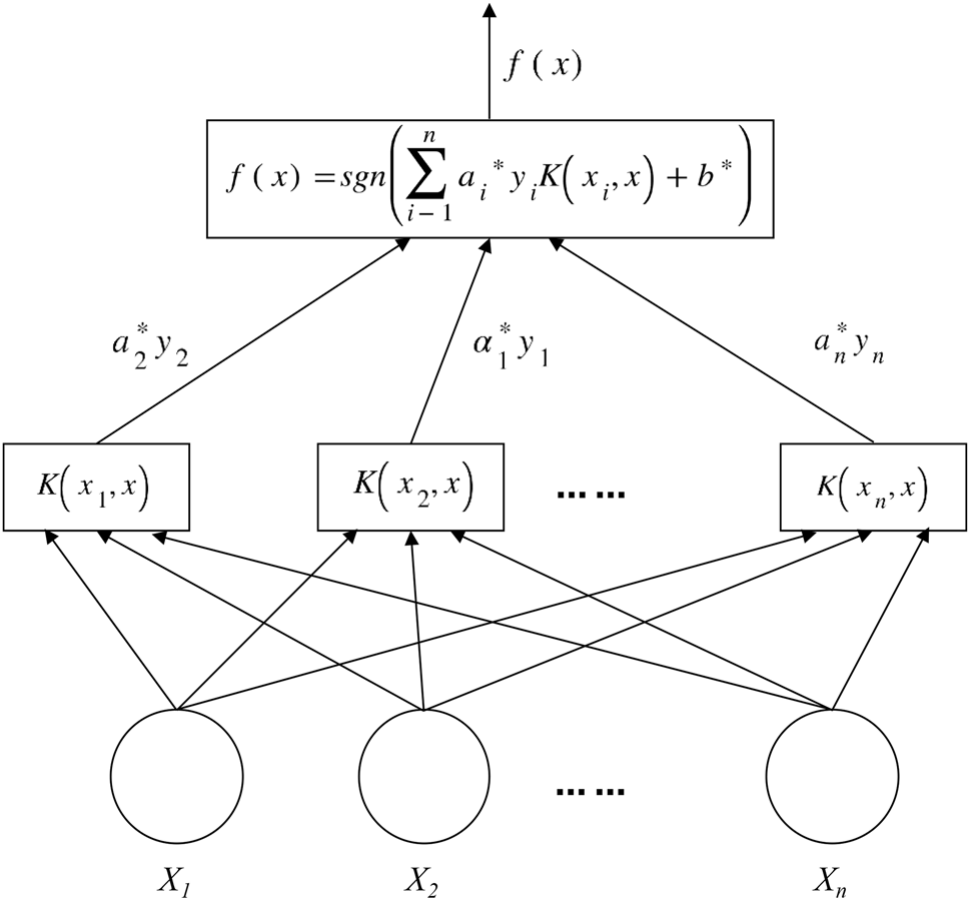
Support vector machine schematic.

Support Vector Machine simplifies complex problems by establishing nonlinear mapping relationships is good at dealing with nonlinear complex systems, and automatically establishes the mapping relationship between water quality indicators and evaluation criteria by performing inner product operations in the transformation space to achieve the purpose of effectively classifying the categories to which the predicted samples belong. The principle is shown in Fig. 1.

The support vector machine consists of three parts: input layer, intermediate inner product kernel function layer, and output layer. The water source discriminant $X_{1} ,X_{2} ,X_{3} , \cdots ,X_{n}$, which represents the sample feature information, is input into the Support Vector Machine model, and the input variables will be processed by the intermediate inner product kernel function layer to map them into the high-dimensional space to seek the optimal solution. This does not consider the specific mapping relationship in the transformation stretching process, and the discriminant type of the water source is finally output in the output layer after a nonlinear transformation^20^.

The procedure of SVM classification operation is as follows^21,22^:

- ① Determine the input sample variable as $\{ x_{i} \} \subset X = R^{n}$, the output variable as $y_{i} \in Y = \{ 1, - 1\}$.
- ② Select the optimal combination of parameters, where the kernel function is $K\left( {x_{i} ,x} \right) = \varphi \left( {x_{i} } \right) \cdot \varphi \left( x \right)$.
- ③ Solve $\min = \frac{1}{2}\sum\limits_{i = 1}^{L} {\sum\limits_{i = 1}^{L} {a_{i} } } a_{j} y_{i} y_{j} K\left( {x_{i} ,x_{j} } \right) - \sum\limits_{i = 1}^{L} {a_{i} }$ according to the constraints.
- ④ The optimal solution $a^{*} = (a_{1} ,a_{2} ,a_{3} ,.....a_{n} )$ is obtained from the above calculation.

After dimensioning, assuming a nonlinear mapping $\varphi :R^{d} \to H$, the optimization problem can be transformed into:

10 $$\begin{gathered} \mathop {\min }\limits_{w,b} \frac{{\left\| w \right\|^{2} }}{2} \hfill \\ s.t.y_{i} (w \cdot \varphi (x_{i} ) + b) \ge 1,i = 1,2, \cdots l. \hfill \\ \end{gathered}$$

Introducing Lagrange multipliers yields:

11 $$L(w,b,a) = \frac{1}{2}\left\| w \right\|^{2} - \sum\limits_{i = 1}^{j} {\alpha_{i} } \left[ {y_{i} \left( {w \cdot \varphi (x_{i} ) + b} \right) - 1} \right].$$

The pairwise objective function is:

12 $$\left\{ {\begin{array}{*{20}l} {\max_{n} \sum\limits_{i = 1}^{i} {\alpha_{i} } - \frac{1}{2}\sum\limits_{i = 1}^{l} {\sum\limits_{j = 1}^{l} {a_{i} } } a_{i} y_{i} y_{i} K(x_{i} ,x_{j} )} \hfill \\ {s.k\sum\limits_{i = 1}^{l} {a_{i} } y_{i} = 0} \hfill \\ {a_{i} \ge 0,i = 1,2, \cdots l} \hfill \\ \end{array} } \right..$$

$K(x_{i} ,x) = \varphi (x_{i} ) \cdot \varphi (x)$ is a kernel function that implicitly maps the data and then learns it. To obtain the classification decision function:

13 $$f\left( x \right) = {\text{sgn}} \left( {\sum\limits_{i = 1}^{l} {y_{i} } a_{i} k\left( {x,x_{i} } \right) + b} \right).$$

The soft interval with the introduction of the penalty factor *C* and the relaxation variable $\xi_{i} (\xi_{i} > 0)$ is optimized as:

14 $$\begin{gathered} \min \frac{1}{2}\sum\limits_{i = 1}^{l} {\sum\limits_{j = 1}^{l} {\alpha_{i} } } \alpha_{j} y_{i} y_{j} K(x_{i} ,x_{j} ) - \sum\limits_{i = 1}^{l} {\alpha_{i} } \hfill \\ 0 \le a_{i} \le C,i = 1,2, \cdots l. \hfill \\ \end{gathered}$$

The optimal decision function can be obtained as:

15 $$f(x) = {\text{sgn}} \left( {\sum\limits_{i = 1}^{l} {a_{i} } y_{i} K(x_{i} ,x) + b} \right).$$

### Optimal parameter solving

**Figure 2:**
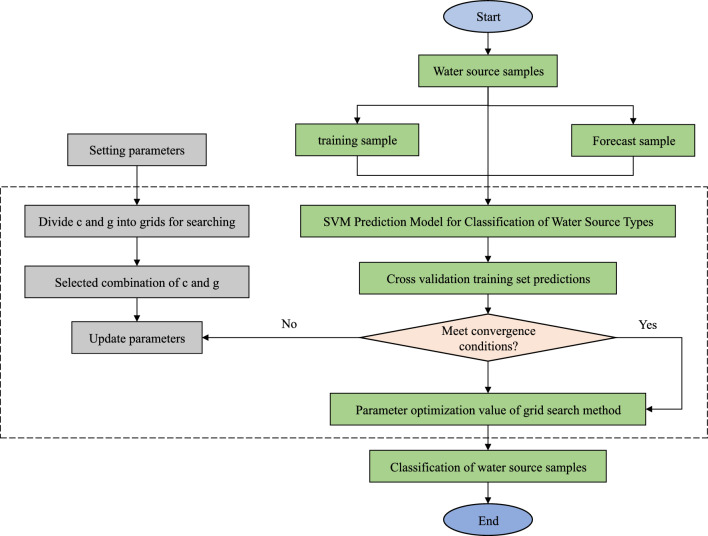
Grid search optimization process.

In this paper, the grid search method is chosen to divide the grid for the optimal search. Using the fixed-step grid search search^23^, a violent search method with a combination of coarse and fine, and a large step size in the optimization search space, all the real target points to be searched are cyclically arranged and combined, and the value range of *c* and *g* are set to [2–10]. The process and principle of the optimization search are shown in Fig. 2.

The support vector machine steps for the optimization of the grid search method are as follows^24,25^:

1. *Create a coordinate grid* Set $$X = \left[ {\begin{array}{*{20}c} {X_{1} ,X_{2} } \\ \end{array} } \right]$$, $$Y = \left[ {\begin{array}{*{20}c} {Y_{1} ,Y_{2} } \\ \end{array} } \right]$$_._ Set up the training learner, pick the step size *L*, put in the parameter search range, and the grid parameter node ${\text{c}} = 2X$,$g = 2Y$.
2. *Using K-fold to find the classification accuracy* The samples are divided into *N* subsets, including the test set and the training set, and the number of subsets is 1 and *N*-1, respectively, where the training set is used for model building. The accuracy evaluation method is set to obtain the classification accuracy corresponding to the set of parameters, which is used for the training set.
3. *Traversing the coordinate grid* The combination with the smallest mean square error among all the traversed parameters is selected to obtain the optimal trainer, that is, the combination of (c, g) with the highest classification accuracy, and the optimal trainer accuracy is output.

## Analysis of water information

### Hydrogeologic conditions in the study area

The coal seams in the Zhaogezhuang Coal mine are predominantly distributed within the Upper Taiyuan Formation (Zhaoge Formation) of the Shanxi Formation (Da Miaozhuang Formation). The presence of faults on the eastern, western, southern, and northern boundaries has resulted in the uplift and exposure of the Ordovician limestone due to tectonic activity. This faulting has led to the development of intense structural karst. Consequently, the gently inclined limestone has formed troughs, and a robust karst development zone has emerged along the eastern boundary fault of the Kaiping block. The overlying Quaternary loose layers exhibit coarse particle size, exceptional permeability, and high water content, serving as a prominent conduit for groundwater movement and constituting the primary strong runoff zone in the regional groundwater system. The hydrodynamic forces are notably strong, displaying characteristics of concentrated conduit flow. Furthermore, a portion of the groundwater in the eastern part of the Shahe River basin in the Zhaogezhuang mine infiltrates the field's interior through the Leizhuang fault, with groundwater flowing from the northeast to the southwest.

The Zhaogezhuang Coal Mine has developed five major aquifer systems from the Cambrian to the Quaternary: the Cambrian aquifer, the Ordovician limestone aquifer, the coal-bearing formation sandstone aquifer, the Tangshan limestone aquifer, and the Quaternary alluvial aquifer.The Quaternary alluvial aquifer in the study area exhibits a relatively thin structure, exerting minimal impact on coal mining operations. In contrast, the Cambrian aquifer predominantly interacts with the Ordovician aquifer. Consequently, the Ordovician aquifer assumes a pivotal role in water influx incidents within the study area, particularly in cases of deep water influx. The principal contributors to these occurrences are the aquifers comprising Ordovician limestone and coal-bearing sandstone within the coal-bearing rock series. To maximize differentiation of water source types, the study selected the six most widely distributed ions in groundwater as discriminative indexes^26,27^. These include Na^+^, Ca^2+^ , Mg^2+^ , Cl^–^, SO_4_^2–^ and HCO_3_^–^. K^+^ was combined with Na^+^ due to their low variation range.

### Data index extraction and collection

For data selection, the Zhaogezhuang mine’s deep mining process was primarily threatened by Ordovician carbonate from the Ordovician aquifer, followed by goaf water damage and sandstone water damage. As a result, four water sample types were chosen: goaf water (from the I aquifer), ordovician carbonate (from the II aquifer), sandstone fracture water from the 13 coal system (from the III aquifer), and sandstone fracture water from the 12 coal seam (from the IV aquifer section A). To screen the typical water sample data, 67 groups were selected from 19 boreholes based on the anion and cation balance test and hydrogeological data of Zhaogezhuang. Among these groups, 18 were from goaf water, 13 from ordovician carbonate, 17 from 13 coal seam sandstone fracture water, and 19 from 12 coal seam sandstone fracture water. The four water sample sources are indicated by I, II, III, and IV respectively. The water samples were submitted to the Testing and Analysis Center of Hebei Coalfield Geology Bureau for chemical analysis. The water quality testing report provided analysis of the main ions and the total hardness (TH) using ion chromatography. Additionally, the bicarbonate ion (HCO_3_^–^) and total alkalinity (TA) were determined through titration using dilute sulfuric acid-methyl orange. The pH value was measured using a pH tester. Subsequently, the data on the nine discriminant indices of the mine water were organized and presented in Table 1(attached).

**Table 1:** 67 groups of water chemistry data.

No	Water sample source	Ion concentration (mmol·L^–1^)								
		Na^+^	Ca^2+^	Mg^2+^	Cl^–^	SO_4_^2–^	HCO_3_^–^	TH	TA	PH
1	I	11.05	66.34	22.23	1.50	81.28	17.21	273.7555	253.71	8.2
2	I	4.36	54.83	40.66	2.68	66.49	30.83	265.32	316.50	8.4
3	I	10.70	54.64	34.18	11.31	70.14	17.54	263.22	398.24	8.2
4	I	5.86	48.45	45.06	1.79	81.84	16.37	256.79	195.19	8.2
5	I	8.91	53.43	37.46	8.45	60.47	31.08	261.92	334.05	8.4
6	I	6.05	54.13	38.71	1.81	76.57	21.62	263.07	187.51	8.2
7	I	5.69	51.70	41.83	1.65	81.41	16.85	257.41	433.20	8.2
8	I	18.02	40.9	40.82	1.75	81.83	16.25	234.65	174.25	8.2
9	I	6.95	44.44	48.09	1.44	85.55	13.00	254.23	192.37	7.6
10	I	2.54	50.85	45.59	2.46	83.76	13.78	264.81	398.24	7.6
11	I	6.09	49.63	43.92	1.58	81.75	16.67	263.97	375.65	8.0
12	I	7.62	49.75	42.40	1.66	81.88	16.46	262.78	421.98	8.2
13	I	7.86	48.59	43.20	1.54	82.39	16.07	260.03	392.07	8.2
14	I	11.81	49.18	37.87	3.08	71.87	21.70	244.56	349.75	8.2
15	I	12.36	52.11	34.83	2.92	72.30	22.78	259.05	398.24	8.1
16	II	6.93	57.86	39.66	4.47	53.01	41.86	249.78	173.56	8.3
17	II	6.52	59.38	40.44	5.61	51.80	41.70	260.07	152.32	8.2
18	II	5.53	60.29	38.49	4.60	50.74	44.62	290.52	164.88	8.0
19	II	12.49	60.85	24.98	12.57	23.62	52.27	283.13	171.39	8.3
20	II	6.26	58.53	39.73	5.19	49.24	42.23	274.09	157.61	8.3
21	II	4.72	61.35	33.83	3.26	48.6	48.13	284.76	159.62	8.3
22	II	6.14	58.45	35.16	3.22	49.56	47.21	263.42	136.55	8.3
23	II	10.46	64.28	24.96	13.06	15.71	60.34	269.77	129.60	8.4
24	II	11.15	67.79	21.06	11.36	19.93	58.08	256.02	115.74	8.3
25	II	11.88	66.20	21.91	13.77	25.31	51.48	248.53	431.240	8.7
26	II	8.8	65.27	24.18	11.16	14.74	61.63	289.71	402.48	8.5
27	III	5.97	54.01	39.52	9.22	13.66	76.64	249.90	378.07	8.7
28	III	8.13	55.92	35.51	9.36	11.97	78.18	253.31	393.04	8.7
29	III	9.74	51.28	38.29	8.45	13.95	76.99	243.85	381.52	8.6
30	III	8.36	52.14	39.12	9.41	13.57	76.96	245.09	393.70	8.7
31	III	13.28	49.67	37.85	10.03	12.43	77.05	209.59	391.27	8.7
32	III	9.85	52.61	36.57	8.44	15.71	75.23	217.28	381.19	8.7
33	III	10.31	51.09	38.00	8.65	14.66	76.08	234.42	329.02	8.4
34	III	9.46	52.49	37.5	8.77	12.88	77.79	241.02	285.95	8.4
35	III	7.71	55.31	36.37	8.67	13.17	77.79	257.60	199.86	8.5
36	III	9.41	50.43	38.87	8.42	14.59	76.60	229.04	367.13	8.3
37	III	12.9	50.31	36.22	9.04	12.34	78.23	214.52	437.58	8.5
38	III	15.11	47.81	36.85	8.4	13.81	77.49	209.10	416.05	8.5
39	III	12.96	48.26	38.24	9.65	13.88	76.17	214.91	349.44	8.5
40	III	9.69	52.50	37.16	9.48	10.86	79.58	216.78	408.70	8.5
41	IV	5.92	61.89	31.72	8.61	15.95	70.99	243.98	319.74	8.4
42	IV	8.35	58.11	33.54	8.75	15.54	71.92	241,75	299.32	8.4
43	IV	9.6	60.39	30.01	8.65	16.38	71.43	259.04	327.90	8.3
44	IV	8.43	58.21	30.85	8.70	14.12	71.22	252,82	259.68	8.0
45	IV	9.27	57.23	30.50	8.77	15.92	70.80	249.18	236.02	8.6
46	IV	8.08	60.91	31.01	8.56	15.44	72.94	261.73	296.56	8.5
47	IV	8.43	59.97	31.55	8.95	16.16	71.80	254.97	280.04	8.4
48	IV	8.16	58.08	33.76	8.87	14.91	72.26	255.03	347.10	8.5
49	IV	5.58	64.36	29.94	8.8	16.42	71.25	271.16	378.85	8.4
50	IV	7.77	57.13	34.88	9.03	13.32	74.16	253.92	279.82	8.0
51	IV	8.80	57.50	33.62	9.94	15.30	70.81	253.18	235.39	8.4
52	IV	4.63	58.01	37.36	10.96	17.03	69.47	256.45	251.13	8.5
53	IV	3.78	61.88	34.32	5.71	16.71	73.42	269.03	366.46	8.4
54	IV	9.4	59.29	30.95	9.54	15.3	71.23	254.01	298.77	8.0
55	IV	2.92	61.30	35.78	9.56	15.85	71.05	265.04	357.90	8.0
56	IV	7.33	60.34	32.33	8.61	16.4	70.84	260.16	400.51	8.4
G1	I	11.77	50.85	36.88	2.51	78.22	19.24	213.24	309.43	8.0
G2	I	10.37	49.73	39.47	2.12	83.91	13.90	213.47	274.06	7.6
G3	I	9.16	50.23	40.17	2.13	82.78	15.09	217.09	209.12	7.9
G4	II	5.87	60.29	38.49	4.60	50.74	44.62	264.55	319.26	8.0
G5	II	10.69	51.17	37.61	9.53	14.63	69.08	221.14	351.53	8.5
G6	III	12.63	47.16	39.19	9.20	14.77	75.28	215.08	376.01	8.3
G7	III	14.49	46.62	37.7	9.41	13.74	76.91	211.75	478.43	8.6
G8	III	1.54	67.1	31.1	8.76	14.58	72.14	290.60	424.92	8.3
G9	IV	11.95	56.99	30.87	9.01	14.93	73.84	247.93	352.32	8.3
G10	IV	6.80	62.73	30.13	8.57	16.88	70.61	267.89	323.04	8.5
G11	IV	12.67	51.17	35.77	10.99	12.71	74.11	231.45	374.40	8.6

**Figure 3:**
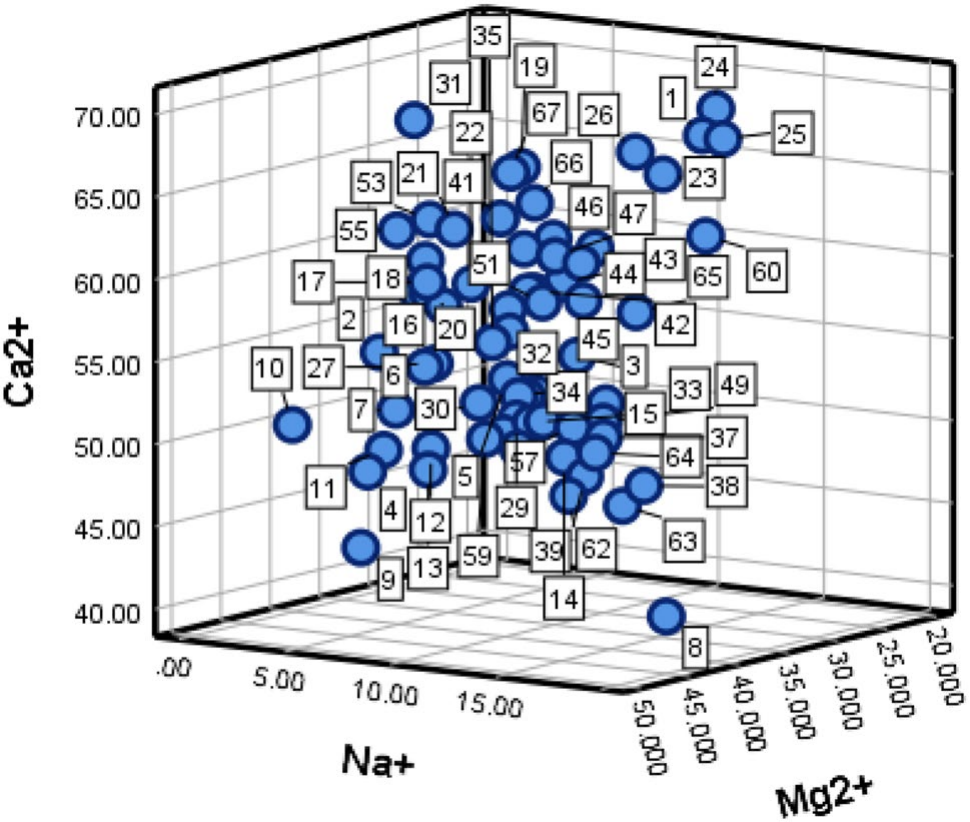
Diagram of anion distribution.

**Figure 4:**
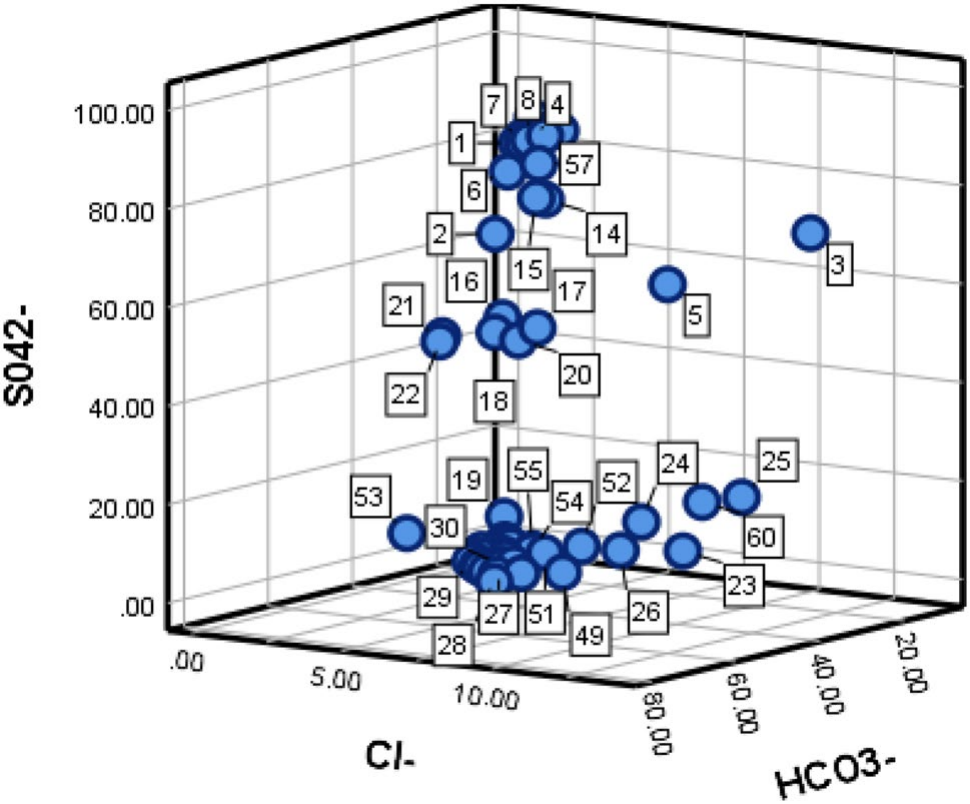
Diagram of cation distribution.

Using 67 sets of typical water sample data collected from the Zhaogezhuang mining area, 56 of these were utilized as training samples for the learning machine as shown in Table 2(attached) while the remaining 11 sets were reserved as test samples, labeled G1 to G11 as presented in Table 3. The distribution of anion and cation content was illustrated using a three-dimensional diagram, with the cation content distribution depicted in Fig. 3, and the anion content distribution shown in Fig. 4.

**Table 2:** Training sample data.

No	Source of water sample	Ion concentration (mmol·L^–1^)					
		Na^+^	Ca^2+^	Mg^2+^	Cl^–^	SO_4_^2–^	HCO_3_^–^
1	I	11.05	66.34	22.23	1.50	81.28	17.21
2	I	4.36	54.83	40.66	2.68	66.49	30.83
3	I	10.70	54.64	34.18	11.31	70.14	17.54
4	I	5.86	48.45	45.06	1.79	81.84	16.37
5	I	8.91	53.43	37.46	8.45	60.47	31.08
6	I	6.05	54.13	38.71	1.81	76.57	21.62
7	I	5.69	51.70	41.83	1.65	81.41	16.85
8	I	18.02	40.9	40.82	1.75	81.83	16.25
9	I	6.95	44.44	48.09	1.44	85.55	13.00
10	I	2.54	50.85	45.59	2.46	83.76	13.78
11	I	6.09	49.63	43.92	1.58	81.75	16.67
12	I	7.62	49.75	42.40	1.66	81.88	16.46
13	I	7.86	48.59	43.20	1.54	82.39	16.07
14	I	11.81	49.18	37.87	3.08	71.87	21.70
15	I	12.36	52.11	34.83	2.92	72.30	22.78
16	II	6.93	57.86	39.66	4.47	53.01	41.86
17	II	6.52	59.38	40.44	5.61	51.80	41.70
18	II	5.53	60.29	38.49	4.60	50.74	44.62
19	II	12.49	60.85	24.98	12.57	23.62	52.27
20	II	6.26	58.53	39.73	5.19	49.24	42.23
21	II	4.72	61.35	33.83	3.26	48.6	48.13
22	II	6.14	58.45	35.16	3.22	49.56	47.21
23	II	10.46	64.28	24.96	13.06	15.71	60.34
24	II	11.15	67.79	21.06	11.36	19.93	58.08
25	II	11.88	66.20	21.91	13.77	25.31	51.48
26	II	8.8	65.27	24.18	11.16	14.74	61.63
27	III	5.97	54.01	39.52	9.22	13.66	76.64
28	III	8.13	55.92	35.51	9.36	11.97	78.18
29	III	9.74	51.28	38.29	8.45	13.95	76.99
30	III	8.36	52.14	39.12	9.41	13.57	76.96
31	III	13.28	49.67	37.85	10.03	12.43	77.05
32	III	9.85	52.61	36.57	8.44	15.71	75.23
33	III	10.31	51.09	38.00	8.65	14.66	76.08
34	III	9.46	52.49	37.5	8.77	12.88	77.79
35	III	7.71	55.31	36.37	8.67	13.17	77.79
36	III	9.41	50.43	38.87	8.42	14.59	76.60
37	III	12.9	50.31	36.22	9.04	12.34	78.23
38	III	15.11	47.81	36.85	8.4	13.81	77.49
39	III	12.96	48.26	38.24	9.65	13.88	76.17
40	III	9.69	52.50	37.16	9.48	10.86	79.58
41	IV	5.92	61.89	31.72	8.61	15.95	70.99
42	IV	8.35	58.11	33.54	8.75	15.54	71.92
43	IV	9.6	60.39	30.01	8.65	16.38	71.43
44	IV	8.43	58.21	30.85	8.70	14.12	71.22
45	IV	9.27	57.23	30.50	8.77	15.92	70.80
46	IV	8.08	60.91	31.01	8.56	15.44	72.94
47	IV	8.43	59.97	31.55	8.95	16.16	71.80
48	IV	8.16	58.08	33.76	8.87	14.91	72.26
49	IV	5.58	64.36	29.94	8.8	16.42	71.25
50	IV	7.77	57.13	34.88	9.03	13.32	74.16
51	IV	8.80	57.50	33.62	9.94	15.30	70.81
52	IV	4.63	58.01	37.36	10.96	17.03	69.47
53	IV	3.78	61.88	34.32	5.71	16.71	73.42
54	IV	9.4	59.29	30.95	9.54	15.3	71.23
55	IV	2.92	61.30	35.78	9.56	15.85	71.05
56	IV	7.33	60.34	32.33	8.61	16.4	70.84

**Table 3:** Forecast sample data.

No	Source of water sample	Ion concentration (mmol·L^–1^)					
		Na^+^	Ca^2+^	Mg^2+^	Cl^–^	SO_4_^2–^	HCO_3_^–^
G1	I	11.77	50.85	36.88	2.51	78.22	19.24
G2	I	10.37	49.73	39.47	2.12	83.91	13.90
G3	I	9.16	50.23	40.17	2.13	82.78	15.09
G4	II	5.87	60.29	38.49	4.60	50.74	44.62
G5	II	10.69	51.17	37.61	9.53	14.63	69.08
G6	III	12.63	47.16	39.19	9.20	14.77	75.28
G7	III	14.49	46.62	37.7	9.41	13.74	76.91
G8	III	1.54	67.1	31.1	8.76	14.58	72.14
G9	IV	11.95	56.99	30.87	9.01	14.93	73.84
G10	IV	6.80	62.73	30.13	8.57	16.88	70.61
G11	IV	12.67	51.17	35.77	10.99	12.71	74.11

### Water chemistry characterization

#### Analysis of statistical characteristic values

The water chemistry statistical characteristic values were calculated and analyzed based on the water chemistry content information of 67 groups of water samples from Zhaogezhuang mine. In the water sample data of study area, the goafwater is obviously different from the other three types of water samples in ionic composition. Among the anions of the goaf water, the anion with the highest content is SO_4_^2–^, which is 78.022 mmol·L^–1^, while the other water samples are HCO_3_^–^. The goaf water is easier to identify than the other three types of water sources, and can be identified by the content of anions, if the highest content of SO_4_^2–^ can be initially classified as goaf water; in the cations, the highest content in all four types of water samples is Ca^2+^. In addition, in terms of the overall content of anions and cations in all water samples data, the content of Ca^2+^  and HCO_3_^–^ is higher compared to other ions, which indicates that Ca^2+^ and HCO_3_^–^ have strong recognition ability.

##### The goaf water

The hydrochemical index of goaf water are as shown in Table 4. The water chemical composition of the four water samples from Zhaogezhuang differed significantly, and their mass concentrations of substances were related to the water source cycle. In the goaf water, the mass concentration of SO_4_^2–^ was the highest in the distribution of anion content, and its substance concentration ranged from 60.47 mmol·L^–1^ to 85.55 mmol·L^–1^, accounting for 78% of the anions, followed by HCO_3_^–^. Cl^–^ had the smallest mass concentration. The cations were mainly Ca^2+^ and Mg^2+^, and the lowest mass concentration of Na^+^. The coefficient of variation is the ratio of the standard deviation to the mean, indicating the degree of dispersion of the data, and the Cl^–^ coefficient of variation was the largest at 0.9, followed by Na^+^  at 0.41, and the rest were smaller, indicating the poor uniformity of ion concentration in the water.

**Table 4:** Hydrochemical index of goaf water.

Index	Na^+^	Ca^2+^	Mg^2+^	Cl^–^	SO_4_^2–^	HCO_3_^–^
Minimum value	2.54	40.90	22.23	1.44	60.47	13.00
Maximum value	18.02	66.34	48.09	11.31	85.55	31.08
Average	8.73	51.09	39.63	2.91	78.02	18.69
Standard deviation	3.63	5.12	5.71	2.63	6.95	5.21
Coefficient of variation	0.41	0.10	0.14	0.90	0.09	0.28

##### Ordovician carbonate

The hydrochemical index of Ordovician Carbonate are as shown in Table 5.The ph of ordovician carbonate is 7.30–7.94, which is weakly alkaline. 86.6% of the anions in ordovician carbonate are mainly HCO_3_^–^ and SO4^2–^, and the mass concentration of cations are: Ca^2+^ > Mg^2+^ > Na^+^, mainly Ca^2+^ and Mg^2+^ accounting for 92.88%, and the water chemistry type is Ca-Mg-HCO_3_. The variation coefficient of ordovician carbonate is in the following order: SO_4_^2–^ > Cl^–^ > Na^+^ > Mg^2+^ > HCO_3_^–^ > Ca^2+^, and the coefficients of variation of all six indexes are less than 0.5. and the coefficients of variation of the anions Cl^–^, SO_4_^2–^, HCO_3_^–^ is greater than that of cations Na^+^, Mg^2+^, Ca^2+^.

**Table 5:** Hydrochemical index of Ordovician carbonate.

Index	Na^+^	Ca^2+^	Mg^2+^	Cl^–^	SO_4_^2–^	HCO_3_^–^
Minimum value	4.72	51.17	21.06	3.22	14.63	41.70
Maximum value	12.49	67.79	40.44	13.77	53.01	69.08
Average	8.26	61.23	31.65	8.15	33.67	52.93
Standard deviation	2.72	4.44	7.43	3.93	16.52	9.70
Coefficient of variation	0.33	0.07	0.23	0.48	0.49	0.18

##### Sandstone fracture water from the 13 coal system

The hydrochemical index of sandstone fracture water from 13 coal system are as shown in Table 6.The highest mass concentration of HCO_3_^–^ among the anions in the fracture water of the 13-coal sandstone is up to 79.58 mmol·L^–1^, the content of SO_4_^2–^ and Cl^–^ is less, and the highest mass concentration of cations is Ca^2+^, followed by Mg^2+^. The 13 coal system sandstone fracture water coefficient of variation is not much different except for Na^+^, which is less than 0.1, and the ion concentration is dispersed more uniformly.

**Table 6:** Hydrochemical index of sandstone fracture water from the 13 coal system.

Index	Na^+^	Ca^2+^	Mg^2+^	Cl^–^	SO_4_^2–^	HCO_3_^–^
Minimum value	1.54	46.62	31.10	8.40	10.86	72.14
Maximum value	15.11	67.10	39.52	10.03	15.71	79.58
Average	10.09	52.04	37.29	9.02	13.56	76.77
Standard deviation	3.36	4.71	1.96	0.49	1.19	1.61
Coefficient of variation	0.33	0.09	0.05	0.05	0.08	0.02

##### Sandstone fracture water from the 12 coal system

The anions in the fracture water of the 12 coal seam sandstone are mainly HCO_3_^–^with a mean mass concentration of 71.79 mmol·L^–1^. The cations are dominated by Ca^2+^ up to 64.36 mmol·L^–1^, followed by Mg^2+^ with a mean concentration of 32.57 and finally Na^+^. The variation coefficients of sandstone fracture water in the 12 coal seam are in the following order: Mg^2+^  > Ca^2+^ > Na^+^  > Cl^–^ > HCO_3_^–^, and the variation coefficient of Mg^2+^ is as high as 0.69.

The hydrochemical index of sandstone fracture water from 12 coal system are as shown in Table 7. In order to study the hydraulic connection between individual aquifers, the degree of connection *K* between them can be calculated quantitatively^28,29^, and since the Cl^–^ concentration is minimally disturbed by other factors and is mainly influenced by the formation itself, the degree of hydraulic connection between two aquifers can be obtained by calculating the difference between their average Cl^–^ concentrations .If the *K* value of the hydraulic connection between the two aquifers is less than 0.2, it means that they have a strong hydraulic connection, if *K* is greater than 0.4, it means that the hydraulic connection between the two aquifers here is weak, if the final calculated *K* value is between 0.2 and 0.4, it means that the hydraulic connection is moderately strong^30,31^.

16 $$K = 0.5 \times \frac{{Cl_{1} - Cl_{2} }}{{(Cl_{1} + Cl_{2} )}}.$$

**Table 7:** Hydrochemical index of sandstone fracture water from the 12 coal system.

Index	Na^+^	Ca^2+^	Mg^2+^	Cl^–^	SO_4_^2–^	HCO_3_^–^
Minimum value	2.92	51.17	29.94	5.71	12.71	69.47
Maximum value	12.67	64.36	37.36	10.99	17.03	74.16
Average	7.78	59.25	32.57	8.97	15.54	71.79
Standard deviation	2.48	2.88	2.25	1.08	1.16	1.31
Coefficient of variation	0.32	0.48	0.69	0.12	0.07	0.02

*Cl*_1_ The average Cl^–^ concentration in aquifer 1. *Cl*_2_ The average Cl^–^ concentration in aquifer 2.

Through Eq. (16), the *K* values of goaf water and Ordovician carbonate, sandstone fracture water of 13 coal system and sandstone fracture water of 12 coal system are all 0.25, and the degree of hydraulic connection is moderate. The *K* value of the hydraulic connection between the goaf water and the sandstone fracture water of 13 coal system is 0.025, and the *K* value of the fracture water with the 12 coal seam sandstone is 0.03, which is a weak hydraulic connection; the *K* value of the fracture water with the 13 coal system sandstone and the 12 coal seam sandstone fracture water is 0.001, which is a very weak hydraulic connection. It can be summarized that there is a certain hydraulic connection between the goaf water and other aquifers, indicating the existence of connection and increasing the difficulty of discrimination.

#### Piper trilinear diagram analysis

The hydrogeological conditions in Zhaogezhuang Coal Mine are characterized by complexity and variability. As demonstrated by the previous analysis of the goaf water composition and other water sources, they exhibit distinguishable differences. To further investigate the distribution patterns of aquifer water samples, the Piper trilinear diagram method was employed for analysis. The ion contents were represented as points on the diagram, allowing for inference of the water chemistry type and quality pattern of the aquifer based on the scatter position of the water samples.

**Figure 5:**
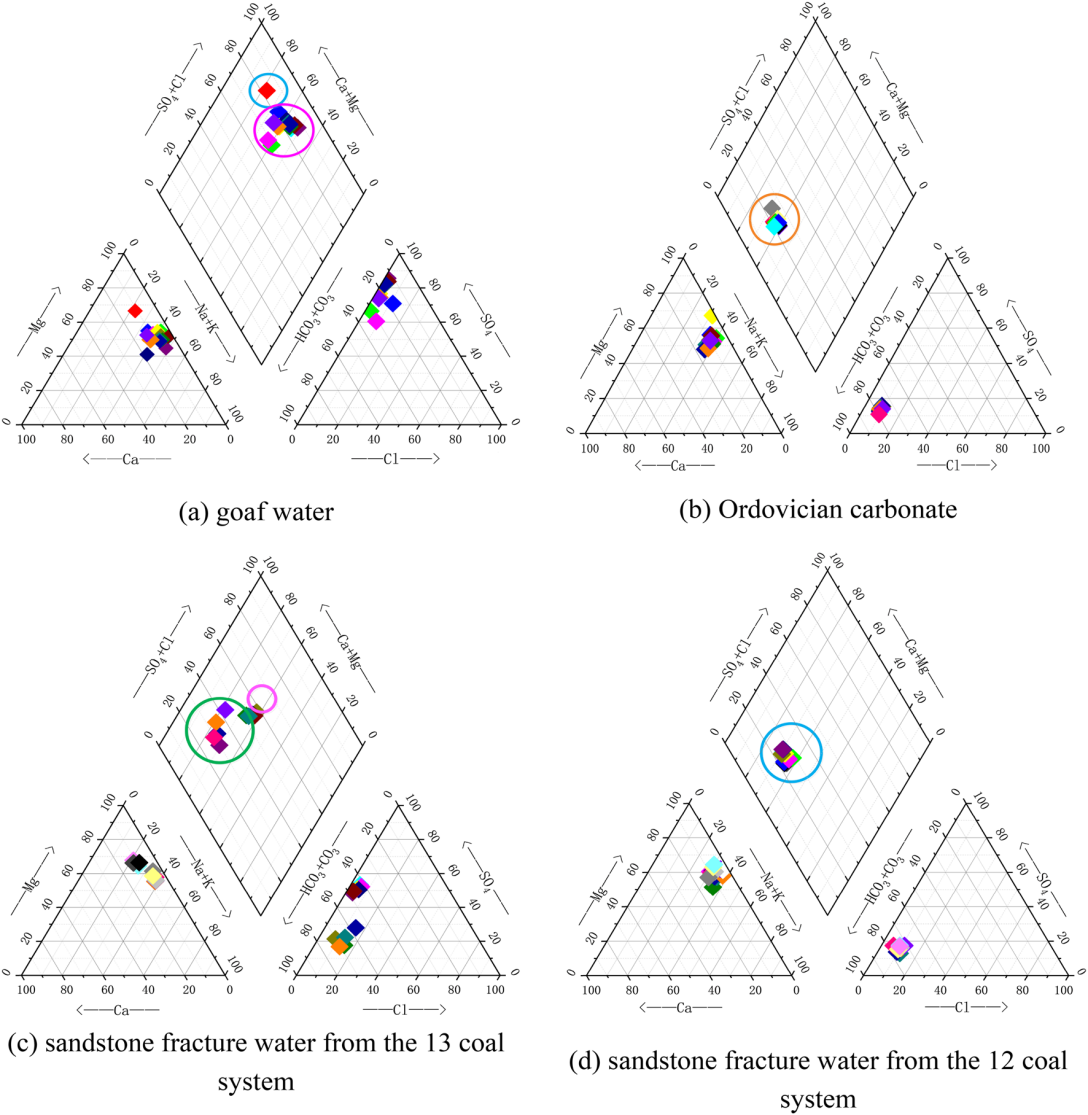
Hydrochemistry analysis trilinear diagram.

The water samples of the study area were drawn for hydrochemistry analysis using piper trilinear diagram shown in Fig. 5. The goaf water was located in the upper right corner, near Ca^2+^, Mg^2+^ and SO_4_^2-^, Cl^–^, mainly Ca·Mg-Cl·SO_4_ type, and individually Ca·Mg-SO_4_ type. The water sample of Ordovician carbonate water is located in the left position of the diamond-shaped area, and the water quality type is Ca·Mg-HCO3 type. By observing the left triangle area, we can find that the cations in the Ordovician carbonate sample are mainly Mg^2+^ and Ca^2+^, and the anions are mainly HCO_3_^–^ and SO_4_^2–^ in the right triangle area. Sandstone fracture water from the 13 coal system is located in the middle and left position, and the cations are mainly located in Ca^2+^ and The anions are scattered in the end elements with high proportion of HCO_3_^–^ and SO_4_^2–^, and the water quality type is Ca·Mg-HCO_3_ type. sandstone fracture water samples from the 13 coal system are highly similar to the 13 in the trilinear diagram, and the water chemistry type is Ca·Mg-HCO_3_ type, the cations are mainly Ca^2+^ and Mg^2+^, and the anions are mainly HCO_3_^–^ and CO_3_^2–^. In summary, the water quality types of Ordovician carbonate, sandstone fissure water from 13 or 12 coal seam are the same, with overlapping characteristics and inconspicuous distribution boundaries, which need further quantitative discrimination.

## Model building and application

### Dimensionality reduction based on R-factor

The normalization process is performed before the operation to make it lie in the interval of [0, 1] to solve the comparability between indicators and ensure the stability of calculation.The normalization of water sample data are as shown in Table 8 (attached).

**Table 8:** Normalization of water sample data.

No	Source of water sample	Ion concentration (mmol·L^–1^)					
		Na^+^	Ca^2+^	Mg^2+^	Cl^–^	SO_4_^2–^	HCO_3_^–^
1	I	0.577	0.946	0.043	0.005	0.942	0.063
2	I	0.171	0.518	0.725	0.100	0.744	0.267
3	I	0.555	0.510	0.485	0.800	0.793	0.068
4	I	0.262	0.280	0.887	0.028	0.950	0.050
5	I	0.447	0.465	0.606	0.568	0.664	0.271
6	I	0.273	0.492	0.652	0.030	0.879	0.129
7	I	0.251	0.401	0.768	0.017	0.944	0.057
8	I	1	0	0.731	0.025	0.950	0.048
9	I	0.328	0.131	1	0	1	0
10	I	0.060	0.370	0.907	0.082	0.976	0.011
11	I	0.276	0.324	0.845	0.011	0.949	0.055
12	I	0.368	0.329	0.789	0.017	0.950	0.051
13	I	0.383	0.285	0.819	0.008	0.957	0.461
14	I	0.623	0.307	0.621	0.133	0.816	0.130
15	II	0.656	0.416	0.509	0.120	0.822	0.146
16	II	0.327	0.630	0.688	0.245	0.564	0.433
17	II	0.302	0.687	0.716	0.338	0.548	0.431
18	II	0.242	0.721	0.644	0.256	0.533	0.474
19	II	0.262	0.884	0.310	0.552	0.134	0.833
20	II	0.286	0.655	0.690	0.304	0.513	0.454
21	II	0.192	0.760	0.472	0.147	0.505	0.527
22	II	0.279	0.652	0.521	0.144	0.518	0.513
23	II	0.541	0.869	0.144	0.942	0.064	0.711
24	II	0.583	1	0	0.804	0.121	0.677
25	II	0.627	0.940	0.031	1	0.193	0.577
26	III	0.440	0.906	0.115	0.788	0.051	0.730
27	III	0.2688	0.487	0.682	0.630	0.037	0.955
28	III	0.399	0.558	0.534	0.642	0.014	0.978
29	III	0.497	0.386	0.637	0.568	0.041	0.961
30	III	0.413	0.417	0.668	0.646	0.036	0.960
31	III	0	0.974	0.371	0.593	0.049	0.888
32	III	0.504	0.435	0.573	0.567	0.064	0.934
33	III	0.532	0.378	0.626	0.584	0.050	0.947
34	III	0.482	0.431	0.608	0.594	0.027	0.973
35	III	0.374	0.535	0.566	0.586	0.030	0.973
36	III	0.477	0.354	0.658	0.566	0.049	0.955
37	III	0.689	0.349	0.560	0.616	0.019	0.979
38	III	0.823	0.256	0.584	0.564	0.039	0.968
39	III	0.692	0.273	0.635	0.665	0.040	0.948
40	IV	0.494	0.431	0.595	0.652	0	1
41	IV	0.265	0.780	0.394	0.581	0.068	0.870
42	IV	0.413	0.640	0.461	0.5928	0.062	0.884
43	IV	0.489	0.724	0.331	0.584	0.073	0.877
44	IV	0.418	0.643	0.362	0.5888	0.043	0.874
45	IV	0.469	0.607	0.349	0.594	0.067	0.868
46	IV	0.396	0.744	0.368	0.577	0.061	0.900
47	IV	0.418	0.709	0.388	0.609	0.070	0.883
48	IV	0.401	0.638	0.469	0.602	0.054	0.890
49	IV	0.675	0.381	0.544	0.774	0.024	0.917
50	IV	0.378	0.603	0.511	0.615	0.032	0.918
51	IV	0.440	0.617	0.464	0.689	0.059	0.868
52	IV	0.187	0.636	0.603	0.772	0.082	0.848
53	IV	0.135	0.780	0.490	0.346	0.078	0.907
54	IV	0.476	0.695	0.365	0.656	0.059	0.874
55	IV	0.083	0.758	0.544	0.658	0.066	0.871
56	IV	0.351	0.722	0.416	0.581	0.074	0.086
G1	I	0.620	0.370	0.585	0.086	0.901	0.093
G2	I	0.535	0.328	0.681	0.055	0.978	0.013
G3	I	0.462	0.346	0.706	0.055	0.962	0.031
G4	II	0.664	0.741	0.145	0.902	0.170	0.589
G5	II	0.555	0.381	0.612	0.656	0.050	0.842
G6	III	0.672	0.232	0.670	0.629	0.052	0.935
G7	III	0.785	0.212	0.615	0.646	0.038	0.959
G8	III	0.712	0.326	0.621	0.696	0.021	0.962
G9	IV	0.631	0.598	0.362	0.613	0.054	0.913
G10	IV	0.319	0.811	0.335	0.578	0.080	0.865
G11	IV	0.245	0.872	0.328	0.596	0.074	0.874

There is a non-linear association between the indicators, and to reduce the correlation between the data, the optimal number of common factors for the six indicators of sodium ion, calcium ion, magnesium ion, chloride ion, sulfate ion, and bicarbonate ion was determined to be 3, denoted as Y1, Y2, and Y3. SPSS software was used to analyze 67 groups of samples and 6 evaluation indicators of Zhaogezhuang based on the correlation calculation steps of R-type factors. The eigenvalues and contribution rates of the main factors were as Table 9.

**Table 9:** Characteristic values and contribution rates of main factors.

Principal factor	Initial eigenvalue			Extracting the eigenvalues of the sum of squares		
	Total	Variance contribution rate (%)	Accumulated contribution rate (%)	Total	Variance contribution rate (%)	Accumulated contribution rate (%)
Y_1_	3.42	56.998	56.998	2.72	45.408	45.408
Y_2_	1.45	24.165	81.163	81.16	30.961	76.369
Y_3_	0.93	15.498	96.660	1.21	20.291	96.660

The cumulative contribution rate of the first three principal factors reaches 96.660%, which indicates that the factors extracted by dimensionality reduction contain 96.660% of the information of the original index data. When the cumulative contribution rate reaches 80%, it shows that the extracted principal factors are reasonable and effective, which indicates that these three principal factors cover most of the water chemistry information and can effectively replace the original indexes.

The factor correlation matrix is as follows:

17 $$A = \left[ {\begin{array}{*{20}c} {1.000} & { - 0.416} & { - 0.167} & {0.231} & { - 0.104} & {0.080} \\ { - 0.416} & { - 1.000} & { - 0.799} & {0.393} & { - 0.362} & {0.286} \\ { - 0.167} & { - 0.799} & {1.000} & {0.589} & {0.000} & { - 0.379} \\ {0.231} & {0.393} & { - 0.589} & {1.000} & { - 0.866} & {0.79} \\ { - 0.104} & { - 0.362} & {0.480} & { - 0.899} & {1.000} & { - 0.987} \\ {0.080} & {0.286} & { - 0.379} & {0.791} & {0.987} & {1.000} \\ \end{array} } \right].$$

The correlation coefficient above 0.8 indicates a strong correlation, while between 0.3 and 0.8 indicates a moderate correlation, and below 0.3 indicates no correlation. The correlation coefficient between Na^+^  and Ca^2+^  is − 0.416, indicating a weak correlation, while with Mg^2+^  is − 0.167, with Cl^–^ is 0.231, with SO_4_^2–^ is − 0.104, and with HCO_3_^–^ is 0.080, all of which have no correlation. The correlation coefficient between Ca^2+^ and Mg^2+^ is − 0.799, indicating weak correlation between Ca^2+^ and other ions. Similarly, Mg^2+^ is not correlated with Na^+^  and weakly correlated with other ions, while Cl^–^ and SO_4_^2–^ are strongly correlated and SO_4_^2–^ and HCO_3_^–^ are strongly correlated.

Using the maximum variance orthogonal rotation method, SPSS rotates to obtain the rotated component matrices. The factor loading matrix and the rotated component matrix were:

$$Z^{\prime}_{(3 \times 6)} = \left| {\begin{array}{*{20}c} {0.101} & {0.788} & {0.600} \\ {0.622} & { - 0.754} & {0.180} \\ { - 0.755} & {0.333} & { - 0.364} \\ {0.922} & {0.215} & { - 0.010} \\ { - 0.929} & { - 0.221} & {0.182} \\ {0.872} & {0.25} & { - 0.384} \\ \end{array} } \right|\quad Z^{\prime}_{(3 \times 6)} \equiv \left| {\begin{array}{*{20}c} {0.096} & { - 0.053} & {0.990} \\ { - 0.286} & { - 0.892} & { - 0.398} \\ {0.279} & { - 0.933} & { - 0.393} \\ { - 0.838} & { - 0.267} & {0.251} \\ { - 0.873} & { - 0.211} & { - 0.022} \\ {0.978} & {0.263} & { - 0.06} \\ \end{array} } \right|.$$

The component conversion matrix is:

$$Z^{\prime\prime}_{(3 \times 3)} = \left[ {\begin{array}{*{20}c} {0.835} & {0.548} & {0.055} \\ {0.338} & { - 0.589} & {0.734} \\ { - 0.434} & {0.594} & {0.677} \\ \end{array} } \right].$$

Three new main components Y_1_, Y_2_, and Y_3_ were extracted, and the factor score coefficient matrix based on SPSS operations was as follows:

$$U = \left[ {\begin{array}{*{20}c} { - 0.072} & {0.079} & {0.838} \\ { - 0.108} & {0.521} & { - 0.241} \\ {0.152} & { - 0.608} & { - 0.264} \\ {0.277} & {0.052} & {0.116} \\ { - 0.410} & { - 0.204} & { - 0.128} \\ \end{array} } \right],$$

According to the factor score coefficient matrix, the expressions of the main factors Y_1_, Y_2_, and Y_3_ are:

$$\left\{ {\begin{array}{*{20}l} {Y_{1} = - 0.072X_{1} - 0.108X_{2} + 0.152X_{3} + 0.277X_{4} - 0.410X_{5} } \hfill \\ {Y_{2} = 0.079X_{1} + 0.521X_{2} - 0.608X_{3} + 0.052X_{4} - 0.204X_{5} } \hfill \\ {Y_{3} = 0.838X_{1} - 0.241X_{2} - 0.264X_{3} + 0.116X_{4} - 0.128X_{5} } \hfill \\ \end{array} } \right..$$

The original data of water samples (I), water samples (II), water samples (III), and water samples (IV) from Zhaogezhuang mine were substituted into the model expressions of the three main factors Y_1_, Y_2_, and Y_3_, and the factor score matrices were as follows:

$$\left( \mu \right)_{18 \times 3} { = }\begin{array}{*{20}l} {\left[ {\begin{array}{*{20}l} { - 2.427} \hfill & {2.841} \hfill & {0.952} \hfill \\ { - 1.011} \hfill & { - 0.465} \hfill & { - 1.304} \hfill \\ { - 0.933} \hfill & { - 0.635} \hfill & {1.059} \hfill \\ { - 1.374} \hfill & { - 1.276} \hfill & { - 0.770} \hfill \\ { - 0.621} \hfill & { - 0.066} \hfill & {0.284} \hfill \\ { - 1.476} \hfill & { - 0.164} \hfill & { - 0.689} \hfill \\ { - 1.511} \hfill & { - 0.660} \hfill & { - 0.801} \hfill \\ { - 1.636} \hfill & { - 1.159} \hfill & {2.954} \hfill \\ { - 1.389} \hfill & { - 1.881} \hfill & { - 0.448} \hfill \\ { - 1.349} \hfill & { - 1.170} \hfill & { - 1.730} \hfill \\ { - 1.441} \hfill & { - 1.052} \hfill & { - 0.711} \hfill \\ { - 1.506} \hfill & { - 1.012} \hfill & { - 0.163} \hfill \\ { - 1.518} \hfill & { - 0.835} \hfill & { - 0.234} \hfill \\ { - 1.377} \hfill & { - 0.355} \hfill & {1.104} \hfill \\ { - 1.516} \hfill & {0.228} \hfill & {1.266} \hfill \\ { - 1.609} \hfill & { - 0.066} \hfill & {1.085} \hfill \\ { - 1.699} \hfill & { - 0.414} \hfill & {0.667} \hfill \\ { - 1.623} \hfill & { - 0.492} \hfill & {0.284} \hfill \\ \end{array} } \right]} \hfill \\ \end{array} \left( \mu \right)_{18 \times 3} { = }\left[ {\begin{array}{*{20}c} { - 0.607} & { - 0.156} & { - 0.731} \\ { - 0.497} & { - 0.107} & { - 0.901} \\ { - 0.556} & {0.111} & { - 1.160} \\ {0.280} & {1.199} & { - 0.906} \\ { - 0.464} & { - 0.140} & { - 0.932} \\ { - 0.693} & {0.626} & { - 1.268} \\ { - 0.672} & {0.281} & { - 0.833} \\ {0.373} & {1.885} & {0.727} \\ { - 0.049} & {2.636} & {0.921} \\ { - 0.021} & {2.541} & {1.268} \\ {0.257} & {1.968} & {1.211} \\ {0.085} & {1.734} & {1.449} \\ {0.837} & { - 0.732} & {0.547} \\ \end{array} } \right].$$

$$(\mu )_{17 \times 3} = \left[ {\begin{array}{*{20}r} \hfill {1.074} & \hfill { - 0.886} & \hfill { - 0.960} \\ \hfill {0.947} & \hfill { - 0.2529} & \hfill { - 0.283} \\ \hfill {0.944} & \hfill { - 0.906} & \hfill {0.179} \\ \hfill {1.065} & \hfill { - 0.943} & \hfill { - 0.228} \\ \hfill {0.581} & \hfill {1.069} & \hfill { - 2.249} \\ \hfill {0.812} & \hfill { - 0.581} & \hfill {0.250} \\ \hfill {0.916} & \hfill { - 0.863} & \hfill {0.364} \\ \hfill {0.963} & \hfill { - 0.729} & \hfill {0.105} \\ \hfill {0.910} & \hfill { - 0.410} & \hfill { - 0.428} \\ \hfill {0.963} & \hfill { - 1.004} & \hfill {0.100} \\ \hfill {0.927} & \hfill { - 0.694} & \hfill {1.162} \\ \hfill {0.852} & \hfill { - 0.918} & \hfill {1.804} \\ \hfill {1.005} & \hfill { - 1.054} & \hfill {1.200} \\ \hfill {1.068} & \hfill { - 0.700} & \hfill { - 0.182} \\ \hfill {0.993} & \hfill { - 1.254} & \hfill {1.103} \\ \hfill {0.982} & \hfill { - 1.108} & \hfill {1.684} \\ \hfill {1.030} & \hfill { - 0.890} & \hfill {1.251} \\ \end{array} } \right](\mu )_{19 \times 3} = \left[ {\begin{array}{*{20}r} \hfill {0.538} & \hfill {0.679} & \hfill { - 0.904} \\ \hfill {0.633} & \hfill {0.212} & \hfill { - 0.196} \\ \hfill {0.439} & \hfill {0.826} & \hfill {0.211} \\ \hfill {0.560} & \hfill {0.512} & \hfill { - 0.055} \\ \hfill {0.521} & \hfill {0.499} & \hfill {0.232} \\ \hfill {0.526} & \hfill {0.707} & \hfill { - 0.272} \\ \hfill {0.549} & \hfill {0.595} & \hfill { - 0.146} \\ \hfill {0.669} & \hfill {0.177} & \hfill { - 0.255} \\ \hfill {0.980} & \hfill { - 0.512} & \hfill {1.176} \\ \hfill {0.796} & \hfill { - 0.055} & \hfill { - 0.383} \\ \hfill {0.714} & \hfill {0.188} & \hfill { - 0.011} \\ \hfill {0.933} & \hfill { - 0.242} & \hfill { - 1.267} \\ \hfill {0.458} & \hfill {0.282} & \hfill { - 1.702} \\ \hfill {0.556} & \hfill {0.662} & \hfill {0.174} \\ \hfill {0.805} & \hfill {0.128} & \hfill { - 1.836} \\ \hfill {0.540} & \hfill {0.519} & \hfill { - 0.495} \\ \hfill {0.564} & \hfill {0.480} & \hfill {0.925} \\ \hfill {0.436} & \hfill {0.952} & \hfill { - 0.626} \\ \hfill {0.466} & \hfill {1.077} & \hfill { - 1.002} \\ \end{array} } \right].$$

### R-SVM model establishment

**Figure 6:**
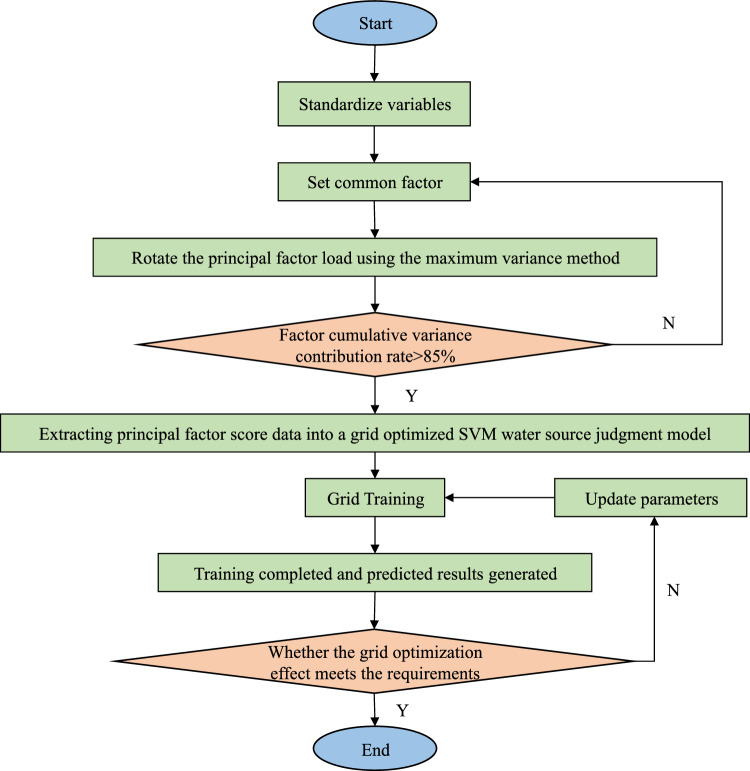
Water source discrimination of R-SVM.

The R- SVM model is shown in Fig. 6. First, the R-factor is used to initially reduce the dimensionality of the data, and the three common factors Y_1_, Y_2_, and Y_3_ are used as the input variables of the model, and the four types of water sources H are used as the output of the model to establish the mapping $F({\text{Y1,Y2,Y3}}) \to H$, which automatically searches for complex connections between the input variables and the types of water sources. The grid search method is used to find the optimal combination of parameters for the Support Vector Machine model. The training set data is then used to train the model, and the trained model is used to predict the water sample types for the testing set data. The predicted types are then compared with the actual types to correct for any deviations. This process is repeated until the model achieves a satisfactory level of accuracy in predicting the types of water samples.

### Parameter search and model application

Six indicators of sodium ion, calcium ion, magnesium ion, chloride ion, sulfate ion and bicarbonate ion are used as input variables of the SVM, and four water source types of goaf water, Ordovician carbonate, sandstone fracture water from the 13 coal system and sandstone fracture water from the 12 coal system are used as outputs of the model to establish the mapping relationship between the two and seek the nonlinear law of the two by SVM. Firstly, 55 sets of training samples and 11 sets of prediction samples are substituted into the grid search method to run the search for parameters, and the range of values of the parameters *c* and *g* of the grid search method are set ${\text{g}} \in \left[ {2^{ - 10} ,2^{10} } \right]$ ${\text{c}} \in \left[ {2^{ - 10} ,2^{10} } \right]$, and the step size *L* = 0.2 according to the operation process of SVM.

**Figure 7:**
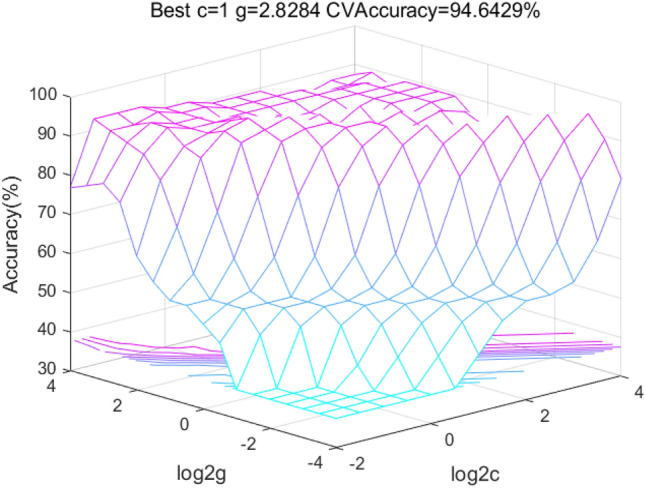
Grid Optimization after dimensionality reduction.

The three public factors of Zhaogezhuang after dimensionality reduction were used as the input variables of the model, and four types of goaf water, Ordovician carbonate, sandstone fracture water from the 13 coal system, and sandstone fracture water from the 12 coal system of Zhaogezhuang mine were used as the outputs of the model to establish the mapping relationship about the public factors and water source types. The factor scores of the 67 sets of sample data after dimensionality reduction were substituted into the SVM model of grid search method for finding the best model for training, and the best parameter combination c = 1 and g = 2.8284 was finally obtained.The result of the optimization search is shown in Fig. 7

**Figure 8:**
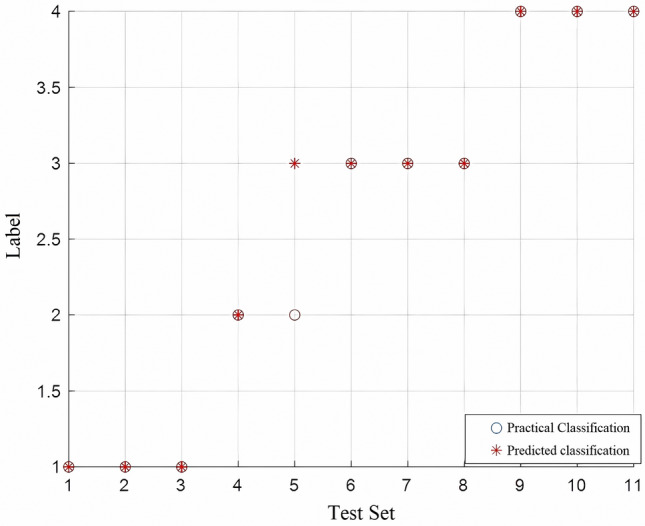
Classification prediction diagram after dimensionality reduction.

Substituting c = 1 and g = 2.8284 into the SVM model, the type attributes were predicted for 11 sets of data to be discriminated, and the final results are shown in Fig. 8 and Table 10. The model misjudged Type II ordovician carbonate as Type III sandstone fracture water from the 13 coal system, indicating that the model is suitable for water source discrimination in Zhaogezhuang Coal Mine and can effectively make the distinction.

**Table 10:** Comparison of model operation results.

	G1	G2	G3	G4	G5	G6	G7	G8	G9	G10	G11
Actual type	I	I	I	II	II	III	III	III	IV	IV	IV
Fisher	I	I	I	IV	IV	III	III	IV	IV	IV	III
Grid	I	I	I	II	III	III	III	III	IV	IV	II
R-type grid	I	I	I	III	III	III	III	III	IV	IV	IV

Table 11 presents a comparative analysis of model performance across different optimization types. The accuracy and precision metrics were employed to evaluate the models' efficacy. The Fisher optimization type exhibits the lowest performance in terms of accuracy and precision. The Grid optimization type shows a significant improvement in both accuracy and precision compared to the Fisher type. Notably, the R-type grid optimization type demonstrates the highest level of performance, surpassing both the Fisher and Grid types in terms of accuracy and precision.

**Table 11:** Comparison of model performance.

Optimization type	Accuracy	Precision
Fisher	63.64%	58.33%
Grid	81.81%	79.17%
R-type grid	90.90%	87.50%

Based on the information provided, it seems that the coupled discriminant model of R-SVM was able to provide more targeted and effective characterization of water sources compared to other multi-model prediction results presented in Table 11. The R-factor simplification was used as a new discriminant to improve the model’s independence component. The coupled discriminant model of R-SVM can also complement the qualitative analysis of water chemistry and provide rapid identification of water sources.

## Conclusion

As coal mine of submarine mining, the identification and prediction of mine water inrush source is of great significance to the safety and efficiency of mine production in Zhaogezhuang Coal Mine. In order to prevent and control the water inrush, it is of great practical significance to identify the mine water source effectively and accurately. Through the analysis of the water source data of different parts in the mine, the effective water source discrimination model was established to verify its effectiveness and practicability.The conclusions of the study are as follows:

1. The chemical composition data of 67 water samples of Zhaogezhuang Coal Mine were collected. According to the chemical composition analysis of selected mine water sources, the main ions identified in water sources were Na^+^, Ca^2+^, Mg^2+^, Cl^–^, SO_4_^2–^ and HCO_3_^–^. The water inrush sources in the mining area were divided into four categories: goaf water was type I, ordovician carbonate was type II, sandstone fracture water from 13 coal seam was type III, and from 12 coal seam was type IV. The analysis and comparison of water source information provide support for the establishment of water source discrimination model.
2. R factor analysis was used to reduce the dimensionality of the original data, resulting in three common factors (Y_1_, Y_2_, and Y_3_) and factor score data for water source data. This approximation of indicator attributes filtered out redundant features and improved efficiency.
3. The coupled model of R-SVM achieved a classification accuracy of 90.90% in water source discrimination for the Zhaogezhuang mine. Compared to traditional qualitative approaches, this model explores the internal laws of the data and provides accurate discrimination, improving upon the Fisher discrimination function and SVM model alone.

## Data Availability

The data used to support the findings of this research are included within the paper.
